# Postprandial cardiogenic syncope caused by gastric polyp-induced pyloric obstruction in an elderly woman with a giant hiatal hernia: a case report

**DOI:** 10.1186/s40792-017-0403-2

**Published:** 2017-12-13

**Authors:** Hideyuki Saito, Tatsuya Miyazaki, Makoto Sohda, Makoto Sakai, Hiroaki Honjyo, Yuuji Kumakura, Tomonori Yoshida, Takehiko Yokobori, Koji Kurosawa, Hiroyuki Kuwano

**Affiliations:** 1 Department of General Surgical Science, Gunma University Graduate School of Medicine, 3-39-22 Showa-machi, Maebashi, Gunma 371-8511 Japan; 2 Department of Molecular Pharmacology and Oncology, Gunma University Graduate School of Medicine, 3-39-22 Showa-machi, Maebashi, Gunma 371-8511 Japan; 3 Department of Clinical Laboratory Center, Gunma University Hospital, 3-39-22 Showa-machi, Maebashi, Gunma 371-8511 Japan

**Keywords:** Hyperplastic polyp, Upside-down stomach, Laparoscopic hiatal hernia repair, Endoscopic polypectomy

## Abstract

**Background:**

Hiatal hernias are common. In some reports, hiatal hernias have been implicated in causing dyspnea, syncope, and heart failure.

**Case presentation:**

An 82-year-old woman with a hiatal hernia was admitted to our hospital because she had experienced postprandial syncope during the last few years. Esophagogastroduodenoscopy revealed a large hiatal hernia and a pedunculated polyp of the stomach antrum that fit into the pylorus. An upper gastrointestinal contrast study showed that the entire stomach had relocated to the thoracic cavity and that the body of the stomach was located above the fundus, resulting in a so-called upside-down stomach. Contrast-enhanced computed tomography revealed that a large portion of the stomach, transverse colon, and part of the pancreas were present in the mediastinum. We then performed transthoracic echocardiography followed by a water pouring test using a nasogastric tube. After instillation of 2000 ml of saline, the left atrium was markedly compressed and the area of the mitral annulus was reduced. We determined that stomach dilation by the hiatal hernia and gastric polyp had caused the syncope. The patient underwent laparoscopic hiatal hernia repair and endoscopic gastric polypectomy, and she experienced no syncopal episodes for 5 months postoperatively.

**Conclusions:**

Clinicians should recognize that a large hiatal hernia may be a risk factor for syncope.

## Background

Hiatal hernias in adults often develop in elderly people, and the main symptoms are heartburn, chest pain, reflux, and dysphagia. Some reports have described cardiac complications such as arrhythmia and heart failure caused by giant hiatal hernias [1, 2]. However, few reports have mentioned heart failure-induced syncope in patients with giant hiatal hernias [3–10]. Reports of such cases are very important to prevent oversight in primary care. No reports have described a syncopal episode caused by a hiatal hernia and prolapsing gastric polyp. We herein document such a case in a patient with a giant hiatal hernia whose chief complaint was loss of consciousness.

## Case presentation

An 82-year-old woman with a hiatal hernia was admitted to our hospital for a detailed examination. She reported repeated postprandial syncope once a year, and the frequency had increased in recent years. She had undergone close examination in other hospitals at the time of the syncopal episodes, and the presence of cardiovascular disease, cranial nerve disease, and endocrine disease was excluded.

**Fig. 1:**
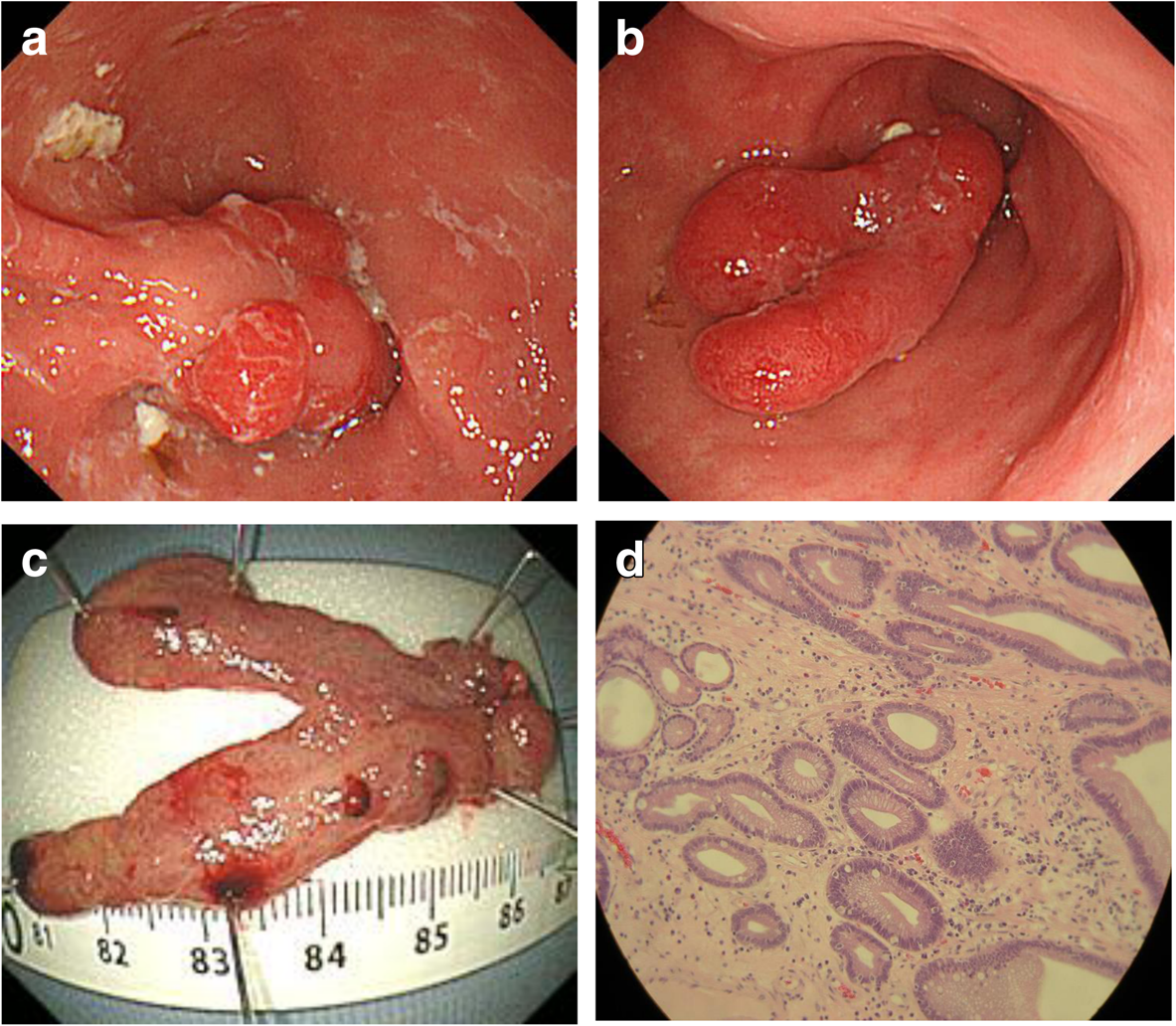
Gastric polyp. Endoscopic examination revealed **a** a pedunculated polyp of the gastric antrum that fit into the pylorus and **b** a pedunculated polyp of the gastric antrum. **c** Polypectomy was performed using a detachable snare over the base of the stalk. **d** Pathological examination revealed a hyperplastic polyp with intestinal metaplasia without malignancy

**Fig. 2:**
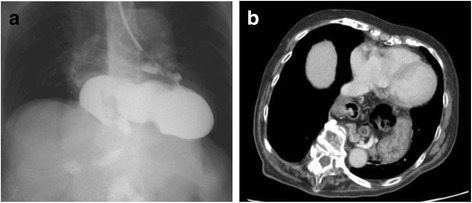
Giant hiatal hernia. **a** An upper gastrointestinal contrast study showed an upside-down stomach. **b** Computed tomography revealed a giant hiatal hernia with the intrathoracic stomach located behind the heart and compressing the left atrium

The patient had a short, stocky build; she was 143-cm tall and had kyphosis. She was undergoing treatment for diabetes mellitus. Hematological examination showed mild anemia. Electrocardiography (ECG) showed sinus rhythm with a heart rate of 64 beats/min and complete right bundle branch block. Esophagogastroduodenoscopy revealed a pedunculated polyp of the stomach antrum that fit into the pylorus (Fig. 1a, b). It showed a mixed type of hiatal hernia but no reflux esophagitis. In the upper gastrointestinal contrast study, the entire stomach had relocated to the thoracic cavity and the body of the stomach was located above the fundus, resulting in a so-called upside-down stomach (Fig. 2a). Contrast-enhanced computed tomography revealed that a large portion of the stomach, transverse colon, and part of the pancreas were present in the mediastinum (Fig. 2b). These structures compressed the left atrium.

**Fig. 3:**
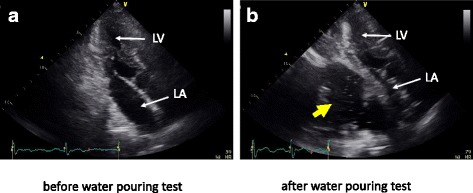
Transthoracic echocardiography. **a** Echocardiography before the water pouring test. **b** Echocardiography showed that after instillation of 2000 ml of saline, the left atrium was markedly compressed and the area of the mitral annulus was reduced. The yellow arrow indicates saline in the stomach

The cause of syncope was suspected to be circulatory failure due to compression by the contents of the hiatal hernia. Therefore, we performed transthoracic echocardiography followed by a water pouring test using a nasogastric tube (Fig. 3). The test involved gradual instillation of saline into the stomach in the sitting position while performing blood pressure, ECG, and echocardiography monitoring. After 2000 ml of saline had been instilled, the left atrium was markedly compressed and the area of the mitral annulus was reduced. No obvious ECG changes or syncope were observed, but a slight decrease in cardiac output was evident.

Surgery with curative intent was performed (laparoscopic hiatal hernia repair and endoscopic gastric polypectomy). The esophageal hiatus was dilated to about 50 mm, which revealed that a large portion of the stomach was present in the mediastinum. The hernia orifice was sutured closed after reduction of the hernia contents. Because this patient showed no evidence of reflux esophagitis, fundoplication was not performed. Finally, we performed polypectomy for removal of the gastric polyps by the usual method using a detachable snare. The postoperative course was good, and the patient was discharged uneventfully. She experienced no syncopal episodes for 5 months postoperatively.

## Conclusions

The patient in this case developed syncope due to heart failure caused by a giant hiatal hernia. Syncope has a variety of causes, including cerebrovascular disease, arrhythmia, hypoglycemia, and autonomic nervous disorders. The vagal reflex is occasionally involved in swallowing. In the present case, we observed narrowing of the left atrium by cardiac ultrasonography. We found that content of hiatal hernia was the cause of the decreased cardiac output.

Including the present case, 10 cases in which a giant hiatal hernia was considered to be the cause of syncope were found in a search of PubMed/Medline from January 1995 to August 2016 (Table 1). The following search terms were used: hiatal hernia OR hiatus hernia AND syncope. Syncope caused by a hiatal hernia seems to be more prevalent in elderly women, with 80% female predominance and a mean age at onset of 79.4 years in women. The main diagnostic tools are computed tomography, ultrasonography, and chest X-ray. Chest X-ray should be considered because a hiatal hernia was diagnosed by X-ray in two reported cases. In most reports, the authors speculated that the syncope was caused by decreased cardiac output due to left atrial retraction. Importantly, the syncopal episodes frequently occurred after a meal. Six of seven patients underwent surgery. The surgical procedures involved hernia repair, reconstruction, and prevention of recurrence. In two reports, the patients underwent laparoscopic fundoplication. Laparoscopic surgery is useful for hiatal hernia repair [11]. In our case, the laparoscopic operation was performed safely and effectively. No mortality occurred in any of these reports.

**Table 1:** Cases with syncope due to a hiatal hernia

Case	First author (year)	Age	Sex	Timing of syncope and accompanied symptoms	Main inspection method	Treatment
1	Akdemir (2001)	70	F	10–15 min after heavy meals	X-ray, CT	Nissen fundoplication
2	Maekawa (2002)	76	F	Immediately after meals	X-ray, CT	Nissen fundoplication
3	Oishi (2004)	76	F	Immediately after meals	US, CT	Not referred
4	Ker (2004)	64	M	Severe chest pain After meals	CT	Laparoscopic Nissen fundoplication
5	Karamitsos (2009)	82	F	After meals	US, CT	Not referred
6	Vanerio (2011)	84	F	After ingestion a carbonated soft drink	CT	Nissen fundoplication
7	Zwermann (2013)	53	M	Chest discomfort	CT	Laparoscopic surgery
8	Gunaruman (2013)	86	F	Nothing	US, CT	Adjustment of diet
9	Our case	82	F	During and immediately after meals	US, X-ray, CT	Hiatal hernia radical operation

We also performed a water pouring test as described by Maekawa et al. [3]. The patient’s vital signs, ECG, blood pressure, oxygen saturation, and consciousness level were monitored to ensure patient safety. The patient was sitting on the chair inserting nasogastric tube, which was placed in the stomach in our study. And we infused water in the stomach step by step observing monitors. We stopped the test when left atrial shrinkage and decreased cardiac output appeared before syncope. We believe that the water pouring test should be very carefully performed with strict monitoring.

Gastric polyps are present in 0.5 to 2.0% of the general population and are usually asymptomatic [12, 13]. Hyperplastic polyps are reportedly the most common type, followed by fundic gland polyps [12]. In the present case, the patient’s gastric hyperplastic polyp prolapsed into the pyloric ring; this conceivably led to failure of gastric emptying, which in turn caused expansion of the stomach in the mediastinum and resultant syncope. We performed simultaneous endoscopic polypectomy and laparoscopic hiatal hernia repair. The current standard treatment strategy for gastric polyps causing obstructive symptoms is complete removal either endoscopically or surgically, followed by pathological analysis. To the best of our knowledge, this is the first report of cardiogenic syncope caused by a gastric polyp accompanied by a giant hiatal hernia.

Parikh et al. [13] analyzed 39 reports of gastric polyps that led to gastric outlet obstruction in the English-language literature. The median size of the endoscopically removed polyps was 3 cm, and the maximum size was 8 cm. The maximum size of all polyps, including those removed surgically, was 13 cm. The gastric polyp in the present case was about 6 cm. The failure of gastric emptying led to stagnation of the gastric contents, which is considered to have promoted gastric distension into the mediastinum. Such a situation is likely to cause syncope. Clinicians should recognize that a hiatal hernia is a risk factor for syncope.

In summary, compression of the heart by a large hiatal hernia resulted in dynamic circulatory changes and syncope. The patient’s gastric polyp facilitated this phenomenon by inhibiting gastric emptying.

## Abbreviation

ECG: Electrocardiography
